# The Parasite Load of *Trypanosoma cruzi* Modulates Feeding and Defecation Patterns of the Chagas Disease Vector *Triatoma infestans*

**DOI:** 10.3390/microorganisms10051003

**Published:** 2022-05-10

**Authors:** Francisco Chacón, Antonella Bacigalupo, Bárbara Álvarez-Duhart, Pedro E. Cattan, Rigoberto Solís, Catalina Muñoz-San Martín

**Affiliations:** 1 Laboratorio de Ecología, Facultad de Ciencias Veterinarias y Pecuarias, Universidad de Chile, Santiago 8820808, Chile; fachacon@veterinaria.uchile.cl (F.C.); abacigalupo@uchile.cl (A.B.); barbara.biri@gmail.com (B.Á.-D.); pcattan@uchile.cl (P.E.C.); 2 Programa de Doctorado en Ciencias Silvoagropecuarias y Veterinarias, Campus Sur, Universidad de Chile, Santiago 8820808, Chile; 3 Institute of Biodiversity, Animal Health and Comparative Medicine, University of Glasgow, Glasgow G12 8QQ, UK; 4 Escuela de Medicina Veterinaria, Facultad de Ciencias Médicas, Universidad Bernardo O’Higgins, Santiago 8370854, Chile; 5 Núcleo de Investigaciones Aplicadas en Ciencias Veterinarias y Agronómicas, Universidad de las Américas, Campus Providencia, Santiago 7500975, Chile

**Keywords:** Chagas disease, behavior, feeding, defecation, host–parasite interactions, insect vector, parasite load, triatominae, *Trypanosoma cruzi*, *Triatoma infestans*

## Abstract

*Trypanosoma cruzi* is the causal agent of Chagas disease, a parasitic zoonosis transmitted mainly through the feces of triatomine insects. *Triatoma infestans* is the main triatomine vector of this disease in South America. Previous research has shown that *T. cruzi* infection modifies the behavior of triatomines. We evaluated, for the first time, the effect of parasite load on feeding and defecation behavior, which we quantified by using real-time PCR. The detection time of the host was shorter in infected individuals, and the number of bites increased, while the dejection time was reduced when compared with the non-infected group. A significant correlation between the parasite load and the behavioral changes registered in the infected triatomines was found. These results would indicate that the intensity of *T. cruzi* infection modulates the feeding and defecation behavior of *T. infestans*, increasing the vector competence of this triatomine vector.

## 1. Introduction

Parasites can cause a wide range of behavioral modifications in hosts [1]. Performance modifications of vector feeding appear to be a common strategy to improve parasite circulation in many host–hematophagous vector associations [2]. Chagas disease is caused by the flagellated protozoan *Trypanosoma cruzi* (Chagas, 1909) (Trypanosomatida: Trypanosomatidae), which is transmitted mainly through the dejections of triatomine insects (Hemiptera: Reduviidae), commonly known as kissing bugs [3]. Triatomines are primarily hematophagous, feeding on the species available in their habitat, and their most studied behavior relates to orientation towards the host, ingestion of blood, and defecation [4,5]. In triatomines, the search for a blood meal is related to the detection of the host’s main signals, odors and heat [6,7], activating their appetitive search and orientation. Water vapor constitutes a short-range orientation cue [8] along with other chemical signals, especially carbon dioxide, which can stimulate triatomines to be attracted to and follow a concentration gradient [9,10]. Odors and heat are also used by triatomines to locate the host’s blood vessels [6,7]. *Triatoma infestans* (Klug, 1834) is a triatomine insect that feeds more quickly than other triatomine species, with higher rates of blood ingestion, resulting in better blood-feeding efficiency [11]. Additionally, *T. infestans* is one of the triatomine species that is characterized by defecating during or shortly after blood ingestion [12].

*Trypanosoma cruzi* infects the host through skin wounds, such as those caused by triatomine bites, directly through mucous membranes [13], and by oral transmission due to contamination of food and drinks with triatomines or their dejections, the latter mode of transmission being increasingly reported in certain areas of Latin America [14]. The vectorial transmission of the parasite to vertebrates would be impaired if the vector leaves the host before defecating, so the time that the triatomine takes to excrete its first dejection is important from an epidemiological point of view [15,16]. Prolonged feeding increases the contact time between the host and the vector, increasing the probability of *T. cruzi* acquisition by the triatomine contact between the parasite and the host [16]; its downside is the enhanced chance of predation by the host of the vector, which in turn could result in oral transmission of *T. cruzi* [17].

Previous evidence shows that triatomines exhibit changes in feeding and defecation behavior that is associated with their infection status. *Trypanosoma cruzi*-infected *T. infestans* defecate in half the time and in greater quantity than non-infected ones [15]. Infected *Mepraia spinolai* (Porter, 1934) detects the host in half the time, increases the number of bites, and defecates earlier than in non-infected individuals [18]. In contrast, *T. cruzi*-infected *Rhodnius prolixus* (Stal, 1859) nymphs feed less frequently than non-infected nymphs [19]. In *R. prolixus*, *Triatoma pallidipennis* (Stal, 1872), *Triatoma longipennis* (Usinger, 1939), *M. spinolai,* and *T. infestans,* there are also reports on modification of their locomotor behavior [20,21,22,23,24].

Classically, *T. cruzi* load was quantified by counting parasites in a Neubauer chamber with a microscope. In a study comparing two groups of *T. pallidipennis* that were infected with different strains of *T. cruzi*, differences were observed in the parasite load, fertility rates, and body size [25]. Another study that quantified *T. cruzi* by Neubauer counting observed that *T. infestans* singly infected with epimastigotes or trypomastigotes of four different strains of *T. cruzi*, belonging to three discrete typing units (DTU), resulted in different parasite loads for each strain throughout the digestive tract [26]. The use of molecular assays based on a quantitative technique such as real-time PCR allows for a more sensitive analysis than conventional parasitological techniques, since it is capable of specifically amplifying the parasite DNA sequence [27,28]; the highly conserved satellite DNA is used as a target for amplification in *T. cruzi*, providing accurate measurements of the parasite load [29,30]. Using real-time PCR, the *T. cruzi* load was quantified in each segment of the intestine of *R. prolixus*, showing differences in the loads depending on the days post-infection: After two weeks, the load in the anterior midgut decreased to a few dozen parasites; in contrast, in the posterior midgut and in the hindgut, the loads remained constant at 10^3^ and 10^4^ parasite equivalents, respectively [31]. Likewise, an increase in *T. cruzi* parasite load was observed by performing real-time PCR in fifth instar nymphs of *R. prolixus,* depending on antioxidant treatments, showing a significant increase in the posterior midgut and rectum when using N-acetylcysteine and urate [32]. *Trypanosoma cruzi* load has also been quantified from dejection samples of xenodiagnosis of *T. infestans* by real-time PCR, evaluating the groups of microscopically negative and positive xenodiagnosis samples separately, obtaining on average 15 and 752 par-eq/mL, respectively [33].

*Triatoma infestans* presents high adaptability to the domiciliary environment [3,34]; it is considered to be the main vector of Chagas disease in Brazil, Argentina, Bolivia, Uruguay, Perú, Paraguay, and Chile. In Chile, it is mostly limited to sylvatic environments due to the control of domiciliary vectors by the “Initiative for the Elimination of *T. infestans* in the Southern Cone Countries” [35,36]. Chile, Uruguay, and Brazil have already certified the interruption of the domiciliary vector transmission by this species [37], but it continues to be a relevant Chagas disease vector in other countries without those levels of control.

So far, there are no previous reports of the relation between the parasite load of *T. cruzi* and the feeding and defecation behavior of *T. infestans*. In this study, we evaluated the behavior of the feeding and defecation activity of *T. infestans* according to *T. cruzi* infection status, and we tested if there is a relation between behavioral changes and the parasite load in these vectors.

## 2. Materials and Methods

### 2.1. Triatomine Insects

*Triatoma infestans* specimens were obtained from the Laboratorio de Ecología, Facultad de Ciencias Veterinarias y Pecuarias, Universidad de Chile, where a colony of insects was maintained under controlled environmental conditions (light/dark cycles of 12 h each, 25 ± 2 °C, 70 ± 10% relative humidity and monthly feeding).

### 2.2. Mammal Hosts Infection

Fifteen naturally infected *Octodon degus* (Molina, 1782) (Rodentia: Octodontidae) were used as hosts to generate part of the infected *T. infestans* group, which was authorized by the Bioethics Committee of the Facultad de Ciencias Veterinarias y Pecuarias, Universidad de Chile (protocol N° 18169-VET-UCH). These rodents are frequently found to be infected by *T. cruzi* [38,39,40]. They were captured from reported foci of *T. infestans* and *M. spinolai*, in the Valparaíso region [41], using Rodentrap™ live-traps baited with rolled oats, and provided with cotton bedding, which was authorized by the Servicio Agrícola y Ganadero (SAG permit 3242/2017), and their infection status was confirmed at the time of capture by real-time PCR as described [42], 18 months before this experiment, so that all the individuals were considered as chronically infected, given that they were not exposed to infected vectors during that period.

Three *Mus musculus* (Linnaeus, 1758) (Rodentia: Muridae) of the BALB/c strain, from the Bioterio Central, Facultad de Medicina, Universidad de Chile, were used as acutely infected hosts under laboratory conditions to generate the rest of the infected *T. infestans* group, which was authorized by the Bioethics Committee of the Facultad de Medicina, Universidad de Chile (protocol N° 19262-MED-UCH). The infection process of the mice consisted of an intraperitoneal inoculation of the Dm28c strain (DTU TcI) of *T. cruzi* obtained from cell cultures (Vero cells, 60–70% confluence) as previously described [43], at a concentration of 1000 parasites in 100 microliters of RPMI 1640 medium (Biological Industries™, Beit Haemek, Israel). Infection of the mice was confirmed by the inspection of blood samples by light microscopy using a Neubauer chamber, measuring the concentration and viability of parasites from day 7 post-infection until reaching ranges between 80,000 and 500,000 parasites/mL.

Rodent hosts were kept in mesh-covered acrylic cages, with food and water ad libitum, and wood chips as bedding, with a room temperature of 20 to 25 °C and relative humidity between 40 and 70%.

### 2.3. Triatomine Infection

We fed two groups of *T. infestans* on infected rodents: One group was composed of 39 third instar and 17 fourth instar nymphs that were fed on naturally infected *O. degus*, and the second group was composed of 26 fifth instar nymphs that were fed on experimentally infected *M. musculus*. The hosts were anesthetized during the feeding of triatomines as described [24].

The control group consisted of 26 third instar nymphs fed on four non-infected *M. musculus*. This group was complemented by those individuals that, after feeding on an infected host, did not acquire the infection (as measured by real-time PCR).

Nymphal instars were determined following Brewer et al. [44], without differentiation by sex, since it is only possible to differentiate them visually in alive *T. infestans* adults. Each triatomine was individually maintained in labeled plastic jars with perforated lids, with folded paper as a shelter, within a climatic chamber with controlled environmental conditions: Light/dark cycles of 12 h each, temperature of 27 ± 1 °C, and 70 ± 10% relative humidity.

All triatomine insects used in this study were subjected to a behavioral analysis of their locomotor activity for 24 h, as reported [24]. After that, each insect was returned to its individual plastic jar in the climatic chamber and was maintained there for 5 ± 2 days before starting the feeding and defecation activity experiments.

### 2.4. Feeding and Defecation Activity

**Figure 1:**
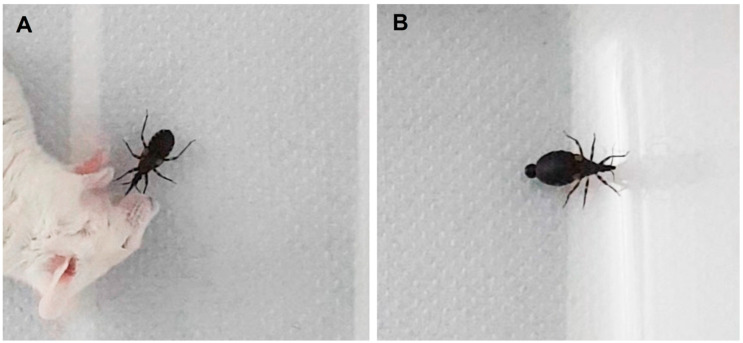
Images of *Triatoma infestans* during feeding and defecation activity. (**A**) Fifth nymphal instar of *T. infestans* biting an anesthetized *Mus musculus* during the recording of feeding behavior activity. (**B**) Fifth nymphal instar of *T. infestans* releasing dejections after the blood intake.

The triatomine behavioral experiments were performed in a square glass experimental arena (24 × 16 × 6.5 cm) provided with white filter paper at the bottom. Experiments were performed by maintaining 25 ± 2 °C, 40 ± 10% relative humidity, were always performed during the morning (9–11 a.m.), by keeping a distance of more than 1 m from the experimental arena, and by always using mask, gloves, goggles, and appropriate clothing to minimize the interaction with the insects. Each triatomine was weighed on an analytical balance (±0.0001 g), before being introduced into the experimental arena. Non-infected white *M. musculus* were anesthetized with ketamine (100 mg/kg)/xylazine (10 mg/kg) and used as a blood source [45]. One mouse was positioned at the center of the experimental arena. The starting point of the triatomine in the arena was randomly assigned among nine quadrants before each of the experiments. After a 2 min period of habituation of the triatomine, in which the insect remained movement-restricted on the arena using a 3 cm diameter opaque tubular container, the container was removed, marking the start of the recording. The movements of the insect were recorded on video until the triatomine deposited the dejection (Figure 1) or until 60 min were completed, whichever occurred first. The triatomine was weighed again and returned to its labeled container. The video record was captured to 30 FPS at a resolution of 1080 pixels using a Sony CX130 camera, with an indirect light source (40 w bulb–65 lux) to avoid shadows, and frame-by-frame analysis was performed in Windows 10 © (Microsoft Corp, Redmon, WC, USA) with Vegas pro 18 ^®^ (Sony Corp, Tokyo, Japan) and VLC media player 3.0 ^®^ (VideoLAN Org, Paris, France) software. During the recording, the same person was present near the arena, always in the same location, and observing the experiment, to monitor both the mouse anesthesia and the duration of the experiment.

Based on methods previously used in the analysis of feeding and defecation activity patterns of *M. spinolai* and *T. infestans* [15,18], the following data were obtained:

Regarding the feeding activity, the variables measured were:

(I) Host detection: Time elapsed—in seconds—since the triatomine begins to mobilize in the arena until the moment when the insect starts walking towards the host;

(II) First approach: Time elapsed—in seconds—between the moment when the insect starts walking towards the host and when it takes the first bite, i.e., pierces the skin of the mouse with the stylet;

(III) Number of bites: Total number of bites performed;

(IV) Feeding time: Time elapsed—in seconds—between the first bite and the end of blood intake of the last bite, including the intervals between bites, when the vector detached from the host.

(V) Weight difference: Subtraction of the initial weight (before starting the experiment) from the final weight (after dejection or at 60 min from the start, whichever occurred first), in milligrams.

The variables regarding the defecation activity were:

(VI) Distance of the dejection: Distance to the host of the released dejection, in centimeters.

(VII) Defecation time: Time elapsed—in seconds—between the end of the blood intake and the start of the dejection release.

### 2.5. Quantification of Trypanosoma Cruzi DNA

The triatomines were euthanized by freezing immediately after finishing the feeding and defecation experiment. DNA extraction, detection, and quantification were performed as previously described [24]: *T. cruzi* was quantified using its nuclear satellite DNA as a target, with primers Cruzi 1 (5′-ASTCGGCTGATCGTTTTCGA-3′) and Cruzi 2 (5′-AATTCCTCCAAGCAGCGGATA-3′) [28]; the endogenous control targeting the conserved region of rDNA of *T. infestans* was quantified using primers 18S For (5′-TCCTTCGTGCTAGGAATTGG-3′) and 18S Rev (5′-GTACAAAGGGCAGGGACGTA-3′ [46].

### 2.6. Statistical Analyses

Each variable was tested for normality using the Kolmogorov–Smirnov test and for homogeneity of variance using the Levene test. We used the Kruskal–Wallis test and the Dunn test for pairwise multiple-comparisons, adjusted by the Bonferroni correction for detecting differences among nymphal instars. We applied the Mann–Whitney U test to analyze the differences between infected and non-infected *T. infestans*. We assessed the correlation between the parasite load and each behavioral variable with the Spearman test. For all tests, a 95% confidence interval (α = 0.05) was used in IBM SPSS Statistics v26 software. For figures, outliers were calculated as previously reported in Chacón et al. (2022) [24].

## 3. Results

After applying the Kolmogorov–Smirnov test, the results indicated that the data were not normally distributed, both for the variables measuring feeding activity and defecation activity (Table S1). Subsequently, we applied non-parametric methods.

### 3.1. Infection Status and Parasite Load Quantification

From the 82 *T. infestans* that fed on infected hosts, 26 of those that fed on chronically infected *O. degus* were not infected. These individuals were added to the control group in posterior analyses, as they showed no significant differences when compared to the non-infected *M. musculus*-fed insects (Table S2). A reduction in infectiousness to triatomines has been reported previously for *O. degus* [47]. The 56 *T. cruzi*-positive triatomines presented parasite loads ranging from 0.8 to 4,867,678 parasite-equivalents per mg (par-eq/mg) of *T. infestans* DNA, with a median of 4046.9 par-eq/mg, a quartile one (Q1) of 79.8 par-eq/mg and a quartile three (Q3) of 45,127.5 par-eq/mg. Detailed results for the real-time PCR runs and parasite loads are available in the Supplementary Materials (Table S3).

### 3.2. Feeding Activity of Triatoma Infestans

We compared all the feeding activity data between infected and non-infected individuals (Table 1). The host detection ranged from 0 to 196 s; the first approach ranged from 1 to 3314 s; the number of bites ranged from 0 to 43 events; the feeding time ranged from 108 to 3600 s; the weight difference ranged from 0 to 310 mg. Individual results for the feeding activity of *T. infestans* are available in the Supplementary Materials (Table S3).

**Figure 2:**
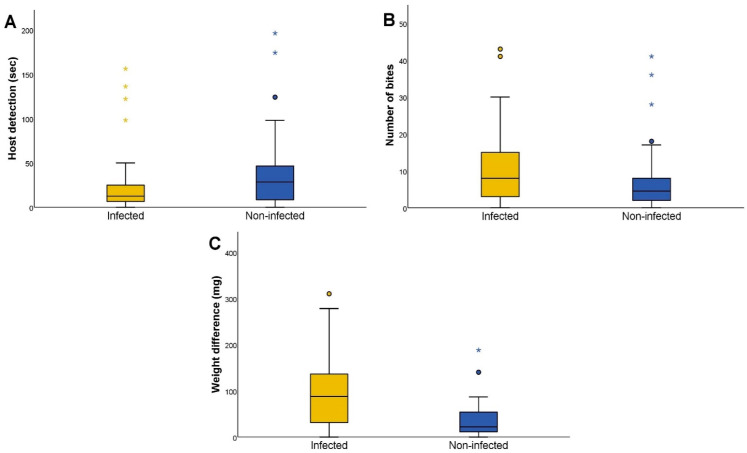
Feeding activity according to the *Trypanosoma cruzi* infection status of *Triatoma infestans*. (**A**) Host detection time, in seconds, of *T. cruzi*-infected and non-infected *T. infestans*. Host detection is defined as the time elapsed since the triatomine begins to mobilize in the arena until the moment when the insect starts walking towards the host. (**B**) The number of bites performed by *T. cruzi*-infected and non-infected *T. infestans*. The number of bites is defined as the total number of bites performed. (**C**) The weight difference, in milligrams, of *T. cruzi*-infected and non-infected *T. infestans*. The weight difference is defined as the subtraction of the initial experimental weight from the final weight. The circles represent outliers and the asterisks represent extreme outliers, calculated as previously reported [24].

We observed significant differences in host detection (Mann–Whitney U test *p* = 0.024), the number of bites (Mann–Whitney U test *p* = 0.025), and weight difference (Mann–Whitney U test *p* < 0.001) according to their *T. cruzi* infection status (Figure 2). Differences were also observed in the rest of the variables, but they were not significant (first approach, Mann–Whitney U test *p* = 0.810; feeding time, Mann–Whitney U test *p* = 0.144).

**Figure 3:**
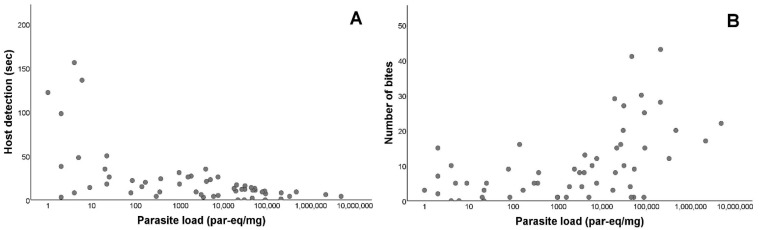
Feeding activity of *Triatoma infestans* according to *Trypanosoma cruzi* parasite load. (**A**) Parasite load (LOG_10_) in relation to host detection time, in seconds. Host detection is defined as the time elapsed since the triatomine begins to mobilize in the arena until the moment when the insect starts walking towards the host. (**B**) Parasite load (LOG_10_) in relation to the number of bites performed by the vector. Number of bites is defined as the total number of bites performed.

Once we established that there were differences in the feeding activity of infected and non-infected *T. infestans*, we evaluated the relationship between the parasite load of *T. cruzi*-positive individuals and the significantly different variables. For the host detection, there was a negative and significant relation (r_s_ = −0.584; *p* < 0.001). Conversely, for the number of bites, there was a positive and significant relation (r_s_ = 0.515; *p* < 0.001; Figure 3). However, there was no significant relations between the parasite load and the weight difference among the infected triatomines (r_s_ = 0.078; *p* = 0.567; Supplementary Figure S1). There were significant differences between the nymphal instars and the weight difference (Kruskal–Wallis test *p* < 0.001), with IV and V instars showing higher blood intake than instar III (Dunn test: III–IV *p* < 0.001; III–V *p* < 0.001; IV–V *p* = 0.299; *p*-values presented were adjusted by the Bonferroni correction). There were no significant differences between the nymphal instars in the rest of the measured variables (Table S4). We also compared the origin of the infection (whether they fed on chronically infected *O. degus* or on acutely infected *M. musculus*), without significant differences being found in any of the variables measured, except in weight difference (Mann–Whitney U test *p* < 0.001; Table S5).

### 3.3. Defecation Activity of Triatoma Infestans

Among all the individuals, only three did not release their dejections during the 60 min period; from these, one was infected and two were non-infected. Regarding the distance of the released dejections to the host, it ranged from 0.2 to 11.9 cm, with no significant differences according to their *T. cruzi* infection status (Mann–Whitney U test *p* = 0.216; Table 2). None of the triatomines released their dejections on the mouse. Individual results for the defecation activity of *T. infestans* are available in the Supplementary Material (Table S3).

**Figure 4:**
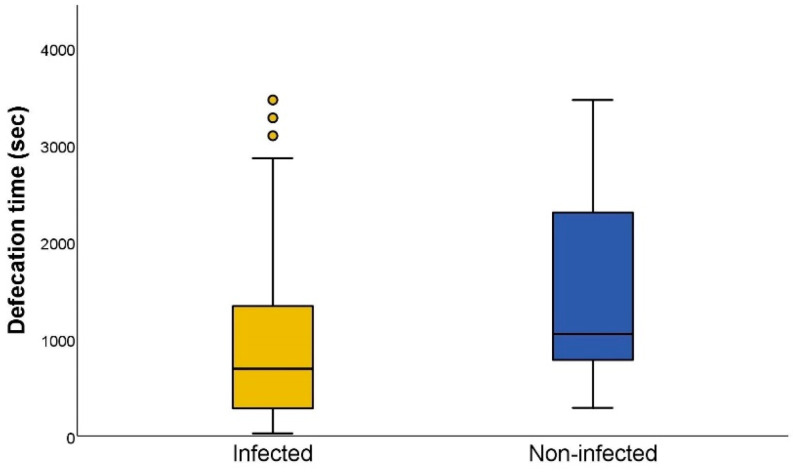
Defecation time, in seconds, of *Trypanosoma cruzi*-infected and non-infected *Triatoma infestans*. Defecation time is defined as the time elapsed between the end of the blood intake and the start of the dejection release. The circles represent outliers, calculated as previously reported [24].

**Figure 5:**
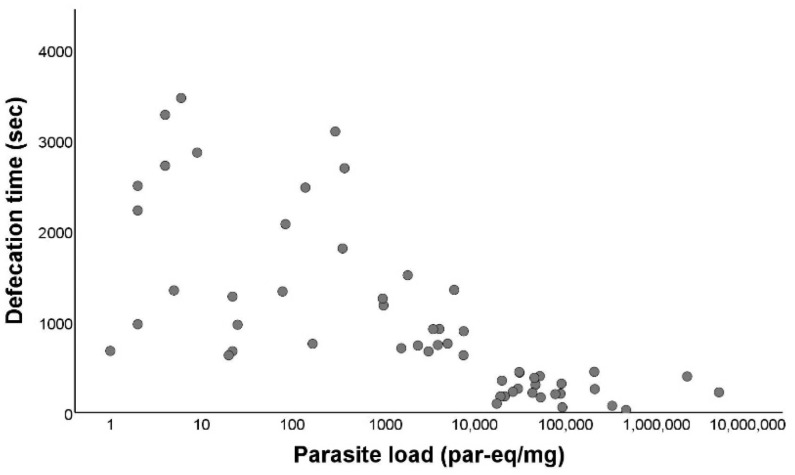
Defecation activity of *Triatoma infestans* according to *Trypanosoma cruzi* parasite load. Parasite load (LOG_10_) in relation to defecation time, in seconds. Defecation time is defined as the time elapsed between the end of the blood intake and the start of the dejection release.

The earliest defecation occurred two seconds after the end of blood intake and the latest happened 3464 s after the end of blood intake. When we compared the defecation time between infected (median: 675 s) and non-infected (median: 1045.5 s) triatomines, the difference between both was significant (Mann–Whitney U test *p* < 0.001; Figure 4). Afterwards, we evaluated the defecation time in relation to the parasite load of *T. cruzi*-positive individuals, showing a negative and significant correlation (r_s_ = −0.779; *p* < 0.001) between the defecation time and the parasite load (Figure 5).

## 4. Discussion

As far as we know, this is the first study where the feeding and defecation behavior of the Chagas disease vector *T. infestans* has been evaluated in relation to its *T. cruzi* load. We performed the analysis of feeding and defecation behavioral variables on 108 *T. infestans*—52 non-infected and 56 infected. We found significant differences between infected and non-infected individuals in the host detection, number of bites, blood ingested, and defecation time. Infected triatomines with a higher *T. cruzi* load showed a decreased host detection time, an increased number of bites, and decreased defecation time.

Behavioral changes have been observed previously in different insect species infected with trypanosomatids, such as *Glossina morsitans* (Wiedemann, 1830) (Diptera: Glossinidae) infected with *Trypanosoma brucei* (Bruce, 1895), which showed more attempts to bite and required more time to ingest blood than for non-infected tsetse flies [48]. *Phlebotomus doboscqi* (Neveu-Lemaire, 1906) (Diptera: Psychodidae) infected with *Leishmania major* (Yakimoff and Schokhor, 1914) has been reported to bite more frequently, but with a lower blood intake than non-infected sand flies [49].

Our results show that the median time of detection of the host by infected *T. infestans* decreases from 28.5, in non-infected individuals, to 12.5 s. We observed that the distribution pattern of the detection time in relation to the parasite load of the infected *T. infestans* showed a decrease in the detection time that was particularly evident in the triatomines with larger *T. cruzi* loads. Previously, it was observed that infected *M. spinolai* nymphs detect the vertebrate host twice as fast as non-infected nymphs [18]. In our work, we observed that the median number of bites increased from 4.5 bites in non-infected triatomines to 8 bites in infected ones, unlike previous studies indicating that *T. cruzi*-infected *R. prolixus* and *T. infestans* did not show differences in bite attempts [15,50]. However, D’Alessandro and Mandel [19] indicated that *T. cruzi-* and *Trypanosoma rangeli*-infected *R. prolixus* increased the number of bite attempts when compared to non-infected triatomines. In infections with *T. rangeli*, this increase in the number of bites of infected *R. prolixus* can be explained by a pathological response in the salivary glands of the triatomine, altering the ability of the vector to find blood vessels and affecting the salivary anti-hemostatic properties [51]; however, as *T. cruzi* only infects the digestive tract of triatomines, this explanation, as far as we know, would not apply in this vector–parasite relation.

As proposed before by several other authors [6,17,21,52,53], a hypothesis to explain the increase in the number of bites and the decrease in host detection time, which was more evident in triatomines with a higher *T. cruzi* load, could be a competition between the triatomine and *T. cruzi* for the nutrients in the ingested blood, leading these insects to a more advanced state of starvation when compared to individuals with a lower parasite load, and those who are non-infected. Triatomines in this state may become more receptive to stimuli: They do not sit and wait until a host appears or move randomly in search of signals; instead, they orient themselves with the air streams to capture smells of interest [7,54]. Another response that may increase due to the lack of nutrients is sensitivity to ammonia that is present in the urine and sweat of hosts, which serves as an attraction factor in *T. infestans* [55]; this response has been measured in the antennae of *R. prolixus*, and it increases during starvation [56]. Finally, another receptive ability that could be increased in infected *T. infestans* is the detection of heat emitted by the host, regardless of its size, even up to two meters away [6,57]. An example of a behavioral change related to the decrease in nutrients promoted by the infection is what was observed in a field study with wild *M. spinolai*, using humans to attract triatomines: A greater number of *T. cruzi*-infected insects were captured in the first hour of exposure; in addition, these vectors had a lower body mass index when compared to non-infected triatomines [52]. This, in turn, would increase the risk of *T. cruzi* infection, given the shorter time that highly parasitized insects take to detect the host and the greater number of skin wounds caused by their bites that could become contaminated with the insect’s dejections [18]. All this evidence together with our results strongly suggest that the parasite lowers the perceptual thresholds of the host.

We also detected a significant difference regarding *T. cruzi* infection status in their weight differences, corresponding to the blood that was ingested and retained by the triatomine after the experiment, with infected *T. cruzi* showing a median weight difference of 88 mg, being 23 mg in non-infected ones. In *M. spinolai*, laboratory individuals infected with *T. cruzi* increased the number of bites, while the amount of blood ingested was not affected by *T. cruzi* infection [18,58]. Verly et al. (2020) [59], observed that *Triatoma rubrovaria* (Blanchard, 1843) infected with *T. cruzi* ingested about 25% more blood than non-infected insects. Our results and others [17] show that there seems to be a taxon-specific effect of the infection, as infected *T. infestans* show the same tendency to increase the number of bites, but also to ingest and retain more blood, unlike other triatomine species. The amount of blood ingested seems to be positively influenced by the *T. cruzi* infection [59]. It is probable that this increase in blood consumption in infected individuals occurs as a way to compensate for the nutrients consumed by the parasites. Previously, it has been documented that the nutritional status of *M. spinolai* individuals infected with *T. cruzi* is lower than that of non-infected individuals [52]. An increase in the intake of infected individuals implies a behavioral change that would favor the transmission of *T. cruzi* to vertebrate hosts [60,61,62,63].

Modification of the defecation pattern is of great importance at the epidemiological level due to the role of this process in the natural transmission of *T. cruzi* to the vertebrate host, where one of the most relevant variables is the time span between feeding and the start of defecation [16]. Our results indicate that the time it takes for infected *T. infestans* to deposit the dejection is shorter—with a median of 675.0 s—than that of non-infected individuals—median 1045.5 s—which agrees with previous studies showing that *T. cruzi* infected nymphs of *T. infestans* defecate in less time; they also documented that the volume of the dejections was greater than those from non-infected ones [15]. Unfortunately, we did not register the volume of the dejection in this study. A similar result than ours was reported for *M. spinolai*, where *T. cruzi*-infected individuals deposited the dejection three minutes earlier than the non-infected ones, on average [18]. In a previous study of infected and non-infected *R. prolixus*, no significant differences were found in the number of bites, feeding time, blood consumed, and defecation time in live host trials [50]. In addition to the infection effect, we observed a significant negative relation of the dejection time with the parasite load, where the higher the load, the less time it took them to defecate. Previously, it has been described that defecation patterns are associated with feeding patterns. The amount of blood consumed is negatively correlated with defecation time and, on the contrary, is positively correlated with the volume of defecation [64,65,66,67]. If *T. cruzi*-positive insects with higher loads consume more blood, they would release increased dejection volumes; on the contrary, those with reduced parasite loads, would ingest less blood, so their dejections would be smaller, which would equate to the blood retained in the infected group regardless of their parasite load, but they would still ingest more than the non-infected group due to their need to incorporate nutrients that are sequestered by *T. cruzi* [68,69,70]. It is possible that this relation explains our findings: We detected a difference in the blood ingested by infected and non-infected individuals, but within the infected, there was no correlation with the parasite load.

The observation that the individuals did not defecate on the mouse when they were fed may be related to the fact that the most accessible points for biting (fewer hairs) and sources of emission of cues used for their detection (temperature, CO_2_, etc.) [6,7,8,9,10] were perceived at the level of the triatomine, in its horizontal plane of displacement.

Regardless of the video system used, it was necessary to complement it with the physical presence of the researcher during recordings because of the anesthetic management and to avoid suffocation of the host. However, the interaction of the responsible re-searcher with the triatomines was avoided as much as possible. Despite this, it is not possible to rule out any effect due to the radiated heat or the CO_2_ expelled by the research-er. Nevertheless, none of the evaluated triatomines directed themselves towards the hu-man present during the experiment (data not shown). In our study, we were only certain of the DTU infecting laboratory mice, but not of those in naturally infected wild rodents. A model of the behavior of triatomines should take into account both the parasite load and its lineage, as this could be useful to predict the behavior of *T. infestans* in areas with different circulating DTUs, which has been described for *T. infestans* [71].

The incongruences observed between our results and other reports, which are focused on the feeding and defecation behavior, could be associated, among others, with the different vector and parasite (*T. cruzi*/*T. rangeli*) combinations or genotypes, their parasite load, their starvation time, time after infection, or different pre-experimental and experimental blood sources [15,16,17,18,19,26,50]. Although these differences make direct comparisons difficult, all of these investigations confirm that *T. cruzi* modifies the behavior of their hosts.

Our results support the hypothesis that the parasite can modify the behavior of the triatomine insect, increasing the transmission rate of *T. cruzi*, which would constitute manipulation of the vector behavior [72]. The observed modifications in behavior reported here, when added to the increased daily activity pattern of infected *T. infestans* described previously by Chacón et al. [24] in the same individuals, could favor the transmission of *T. cruzi* to mammalian hosts, with faster host detection, increased ingestion of blood, which could imply an increased detriment for the host, and more bites that entail more potential wounds for parasite contamination from the dejections, which would be released earlier. In addition, most of these variables would be associated with the *T. cruzi* parasite load of *T. infestans*, where the higher the load, the greater would be the vector efficiency, favorably modulating the vector competence of this species [14,60,73,74].

## Figures and Tables

**Table 1:** Summary values of the feeding activity according to *Trypanosoma cruzi* infection status of *Triatoma infestans*.

	Infection Status	N ^1^	Units	Median	Q1 ^2^	Q3 ^3^
Host Detection *	Infected	56	Seconds	12.5	6.2	25.5
	Non-infected	52	Seconds	28.5	8.3	47.3
**First Approach**	Infected	55	Seconds	9.0	6.0	27.0
	Non-infected	50	Seconds	12.0	6.8	27.1
Number of Bites *	Infected	56	Events	8.0	3.0	15.0
	Non-infected	52	Events	4.5	2.0	8.0
**Feeding Time**	Infected	55	Seconds	872.0	557.0	1187.0
	Non-infected	50	Seconds	711.0	441.8	1051.0
Weight Difference ***	Infected	56	Milligrams	88.0	30.0	138.3
	Non-infected	52	Milligrams	22.1	11.0	54.3

^1^ Number of individuals, ^2^ Quartile 1, ^3^ Quartile 3, Significant difference * *p* < 0.05, *** *p* < 0.001.

**Table 2:** Summary values of the defecation activity according to *Trypanosoma cruzi* infection status of *Triatoma infestans*.

	Infection Status	N ^1^	Units	Median	Q1 ^2^	Q3 ^3^
Dejection Time ***	Infected	55	Seconds	675.0	262.0	1277.0
	Non-infected	50	Seconds	1045.5	765.0	2323.5
**Distance of the** **Dejection**	Infected	55	Centimeters	7.10	3.8	9.6
	Non-infected	50	Centimeters	8.1	4.8	9.9

^1^ Number of individuals, ^2^ Quartile 1, ^3^ Quartile 3, Significant difference *** *p* < 0.001.

## Data Availability

Data is contained within the article or Supplementary Materials.

## Supplementary Materials

The following supporting information can be downloaded at: https://www.mdpi.com/article/10.3390/microorganisms10051003/s1, Figure S1: Weight difference of *Triatoma infestans* according to *Trypanosoma cruzi* parasite load; Parasite load (LOG_10_) in relation to weight difference in milligrams, Table S1: Results of the Kolmogorov–Smirnov test for normality and the Levene test for homogeneity of variance, for each feeding and defecation behavioral variables studied, comparing *Trypanosoma cruzi* infected with non-infected *Triatoma infestans*, Table S2: Results of the Mann–Whitney U test comparing each feeding and defecation behavioral variables studied between non-infected *T. infestans* fed *O. degus* and non-infected *T. infestans* control group, Table S3: Compiled database with all individuals tested for each of the variables analyzed, Table S4: Results of the Kruskal–Wallis test comparing each feeding and defecation behavioral variables studied between nymphal instars (III, IV, and V), Table S5: Results of the Mann–Whitney U test, comparing each feeding and defecation behavioral variable studied between the group of *T. infestans* infected with chronically infected *O. degus* and the group of *T. infestans* infected with acutely infected *M. musculus*.
