# Supplementary material for: The Parasite Load of Trypanosoma cruzi Modulates Feeding and Defecation Patterns of the Chagas Disease Vector Triatoma infestans
Source: Microorganisms. 2022 May 10;10(5):1003. doi: 10.3390/microorganisms10051003 (PMC9143535; doi:10.3390/microorganisms10051003)
Supplement: Supplementary file 1 [file microorganisms-10-01003-s001.zip › Table S1, Table S2, Table S4, Table S5 and Figure S1.pdf]

Table S1. Results of the Kolmogorov-Smirnov test for normality and the Levene test for homogeneity of variance, for each feeding and defecation behavioral variables studied, comparing *Trypanosoma cruzi* infected with non-infected *Triatoma infestans*.

	Kolmogorov-Smirnov	Levene Test
Host detection	p<0.001	p=0.136
First approach	p<0.001	p=0.843
Number of bites	p<0.001	p=0.054
Feeding time	p<0.001	p=0.643
Weight difference	p<0.001	p<0.001
Dejection time	p<0.001	p=0.393
Distance of the dejection	p<0.001	p=0.404

Table S2. Results of the Mann-Whitney U test comparing each feeding and defecation behavioral variables studied between non-infected *T. infestans* fed *O. degus* and non-infected *T. infestans* control group.

	Significance
Host detection	p=0.305
First approach	p=0.712
Number of bites	p=0.423
Feeding time	p=0.415
Weight difference	p=0.969
Dejection time	p=0.380
Distance of the dejection	p=0.838

Table S4. Results of the Kruskal-Wallis test comparing each feeding and defecation behavioral variables studied between nymphal instars (III, IV and V).

	Statistic	Significance
Parasite Load	2.651	p=0.266
Host detection	2.589	p=0.274
First approach	2.754	p=0.241
Number of bites	4.397	p=0.111
Feeding time	2.754	p=0.252
Weight difference	58.685	p<0.001*

Dejection time	5.931	p=0.052
Distance of the dejection	0.714	p=0.700

*Dunn test: III-IV p<0.001; III-V p<0.001; IV-V p=0.299. p-values adjusted by the Bonferroni correction.

Table S5. Results of the Mann-Whitney U test comparing each feeding and defecation behavioral variable studied between the group of *T. infestans* infected with chronically infected *O. degus* and the group of *T. infestans* infected with acutely infected *M. musculus*.

	**Significance**
Parasite Load	P=0.730
Host detection	p=0.948
First approach	p=0.839
Number of bites	p=0.515
Feeding time	p=0.363
Weight difference	P<0.001
Dejection time	p=0.859
Distance of the dejection	p=0.149

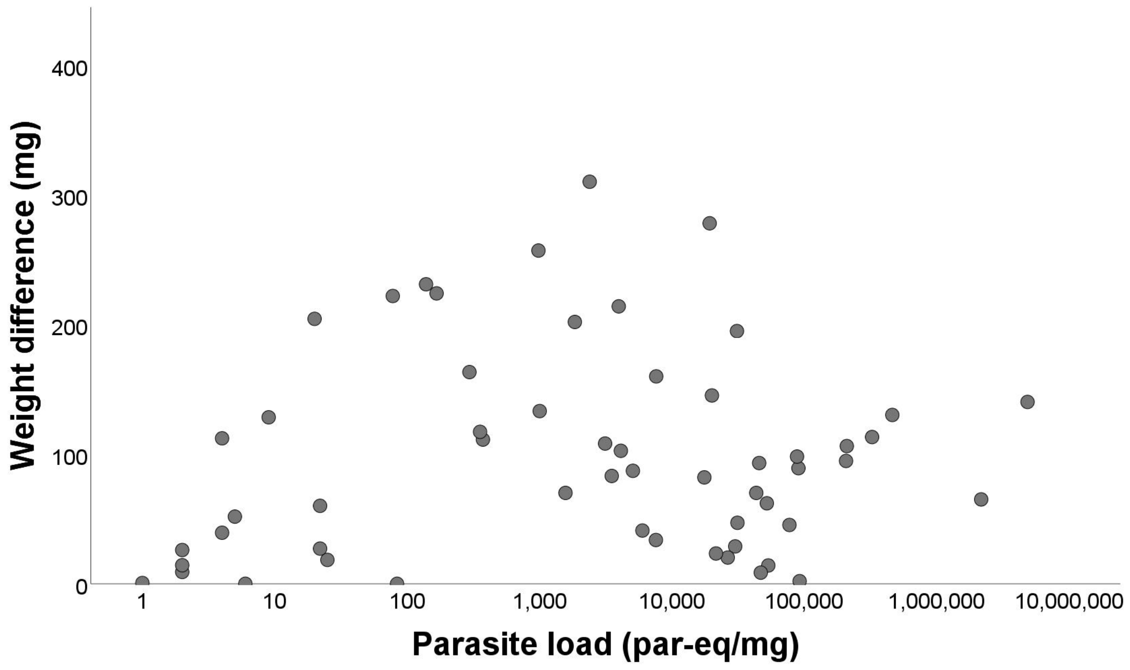

Figure S1. Weight difference of *Triatoma infestans* according to *Trypanosoma cruzi* parasite load. Parasite load (LOG_{10}) in relation to Weight difference in milligrams. Weight difference is defined as the subtraction of the initial experimental weight from the final weight.
